# Horner's Syndrome as a Complication of Ultrasound-Guided Central Cannulation: A Case Report

**DOI:** 10.7759/cureus.28700

**Published:** 2022-09-02

**Authors:** Leonor Silva, Ana Filipa Junqueira, Rita Pato, Sílvia Farraposo, Ana Rita Cruz, Teresa Rocha

**Affiliations:** 1 Anesthesiology, Centro Hospitalar Universitário de Lisboa Central, Lisbon, PRT

**Keywords:** ptosis, postpartum, obstetrics anesthesia, internal jugular vein, horner’s syndrome, catheterization

## Abstract

Cannulation of the internal jugular vein is often necessary for the management of critically ill patients. Despite being a very common procedure and performed more and more safely, several complications still occur. Horner's Syndrome (HS) is one of those complications described before the use of ultrasound as a method of guidance. HS is caused by functional interruption of sympathetic nerve supply to the eye, leading to a classic triad of ipsilateral ptosis, miosis, and anhidrosis. We present the case of a patient, in need of emergent surgery to control the hemorrhagic focus after delivery, with a transient HS secondary to internal jugular vein cannulation under real-time ultrasound guidance.

## Introduction

Cannulation of the internal jugular vein is often necessary for the management of critically ill patients. Despite being a very common procedure and performed more and more safely, several complications still occur [1]. The complications occurring during central venous cannulation are cardiac electrical conduction problems, arterial puncture, pneumothorax, superior vena cava wall perforation, loss of lead wire, Horner's Syndrome (HS), hemothorax, cardiac tamponade, and infection [2,3]. Some are transient, others can even be life-threatening.

Horner's Syndrome (HS) is one of those complications described [4,5]. It is a relatively rare neurologic syndrome characterized by ipsilateral miosis, ptosis, anhidrosis, and enophthalmos due to sympathetic nerve injury or block [6,7]. Risk factors may be repeated attempts of puncture, anatomic landmark technique without ultrasound guidance, accidental carotid artery puncture, or hematoma formation [8]. This syndrome has also been described after epidural catheter placement [9,10].

We are going to present a case of a patient in hemorrhagic shock, who required placement of a central venous catheter and who had Horner's Syndrome as a complication.

## Case presentation

We present a case of a 32-year-old woman with a twin pregnancy, without any relevant past medical history. At 33 weeks, she presented to maternity referring to painful contractility and decreased fetal movements since the same morning. Through ultrasound, one of the fetuses was found dead and placenta abruption was confirmed, so an urgent cesarian section was performed under combined spinal anesthesia. Intraoperatively, a hemorrhage occurred due to placental abruption that was controlled easily. In the end, the epidural catheter was removed after a 10 mL bolus of 0.2% ropivacaine.

**Figure 1:**
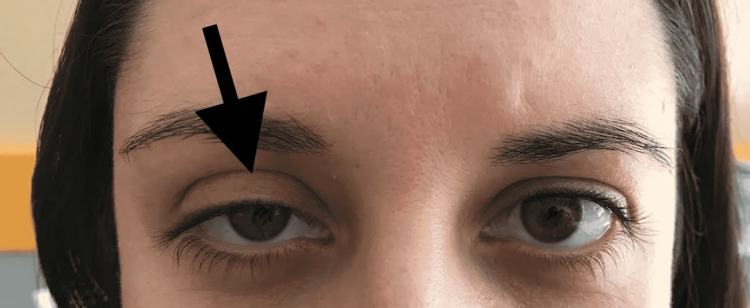
Ptosis and miosis of the right eye

The next day, the patient became weak, with hypotension and tachycardia. On medical evaluation, she presented pallor, decreased capillary perfusion time, and diffuse abdominal pain. Complementary diagnostic tests revealed the presence of hemoperitoneum and severe anemia. She was taken to the operating room for bleeding control. Induction of anesthesia was performed with ketamine 150 mg, propofol 50 mg, and rocuronium 80 mg and was maintained with sevoflurane. An apparently uncomplicated right internal jugular venous catheter was cannulated under real-time ultrasound guidance without the use of local anesthesia. The patient was placed in the Trendelenburg position and with a slight head rotation toward the opposite direction. The short-axis view was used to identify the structures of interest (right internal jugular vein, carotid artery, and the prominent anterior tubercle of the C6 transverse process). Under direct visualization, we introduced a 7Fr triple lumen catheter into the right internal jugular vein using the Seldinger technique. Lumens were tested, and the catheter was positioned correctly. No accidental carotid artery puncture or hematoma formation was noticed. No repeated attempts of puncture were done. Emergence from anesthesia occurred without any problem, and the patient was transferred to the intensive care unit while she was being hemodynamically stabilized. Nearly 12 hours later (24h after removing the epidural catheter), right ocular ptosis, miosis, anhidrosis, and conjunctival hyperemia were noticed by the anesthesiologist (Figure 1).

We contacted a neurologist who recommended that the patient be submitted to a cranioencephalic magnetic resonance and a cervical CT, which she did later the same day. Both were within normal limits. The neurologic and ophthalmologic evaluations were accomplished, both emphasizing the diagnosis of Claude-Horner Syndrome secondary to right internal jugular vein cannulation.

**Figure 2:**
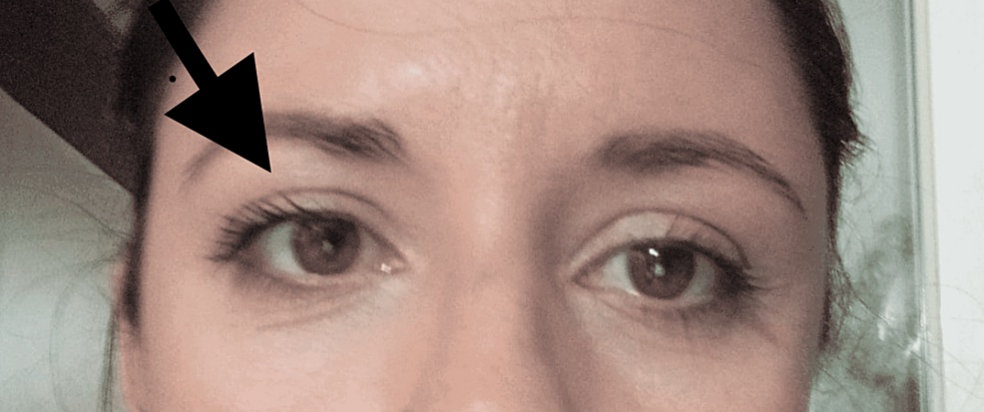
Six months later, no ptosis or miosis of the right eye was noticed

After six months, the ptosis and miosis had disappeared (Figure 2).

## Discussion

The incidence of HS is estimated to be less than 5% after internal jugular vein (IJV) cannulation by anatomical references [4]. It can occur due to damage or blockage along any point in the oculosympathetic pathway (OSP), including at the preganglionic and postganglionic fibers [11]. The cardinal symptoms of Horner Syndrome are drooping of the upper eyelid, decrease in pupil size, and lack of sweat production on a hemiface [12]. Conjunctival hyperemia due to vasodilation of capillaries secondary to sympathetic denervation is also a transient acute sign of HS. The clinical findings can vary based on the location of the lesion in the OSP [11,12].

After the publication of the "Practice Guidelines for Central Venous Access 2020," which recommend the use of ultrasound for central venous catheterization [13], no robust studies were performed to determine the incidence of this complication. There are also no reports of isolated cases with the use of ultrasound.

Despite all of the cervical ganglia supplying postganglionic fibers to the head and neck region, the superior cervical ganglion is the primary contributor via the oculosympathetic pathway [14,15]. Normally, the IJV runs on the anterolateral side of the cervical sympathetic trunk [14]. Risk factors are well-documented: over-rotation of the patient's head, repeated cannulation attempts, anatomical landmark method, accidental carotid artery puncture, or hematoma formation [4,5,8]. The use of high-resolution, real-time, ultrasound-guided puncture would be expected that it would decrease the incidence of OSP trauma, but we cannot say that this happens in clinical practice due to a lack of studies. In our clinical case, we think that it must have been caused by direct trauma from the needle. Over-rotation of the patient's head and the speed in the execution of the technique, given the emergency of the clinical situation, were the probable causes of this outcome. If we had used a local anesthetic to infiltrate the skin, a possible cause would be the dispersion of the local anesthetic, although this is unlikely given the low volume used. Another possible cause is an injury to the longus colli muscle, which is closely related to the stellate ganglion.

It is also important to mention that the patient had had an epidural catheter placed hours before. It has been documented in the literature that HS with an epidural is more likely to occur in pregnant patients due to the physiological and anatomical changes that can occur during pregnancy, especially due to the high cephalad spread of local anesthetic [16,17]. We exclude the epidural as a cause of HS once it was removed 24 hours before the onset of HS. Cervical plexus fiber blockade due to epidural drug administration starts after an average of 27 minutes [18]. The drug used 24 hours earlier was ropivacaine, which has an average mean absorption t1/2 of approximately 4.2 hours so it could not be the cause of HS [19].

## Conclusions

Horner Syndrome associated with IVJ cannulation is rare, usually benign, and transient. This case alerts us to three things. The first is that despite the use of ultrasound, we cannot rule out that this type of complication may arise. The second is that there may be more possible causes and they should be ruled out. The third is that we must perform cannulation as a maximum of safety, avoiding sudden movements and advances of the needle in order to minimize complications. The fact that the symptoms persisted for six months supports the authors' idea that Horner Syndrome was caused by injury to the cervical sympathetic nerve itself or its surrounding tissue during puncture. No definitive treatment exists, meaning that prevention is very important.
